# P37 A retrospective audit of molnupiravir prescribing in the virtual hospital via the CMDU pathway

**DOI:** 10.1093/jacamr/dlad066.041

**Published:** 2023-06-14

**Authors:** Christiana Ogunmodede, Beryl Alexis Lo

**Affiliations:** Royal Berkshire NHS Foundation Trust; Royal Berkshire NHS Foundation Trust

## Abstract

**Background:**

In December 2021, COVID-19 medicine delivery units (CMDU) were launched across NHS hospitals in England. They offered treatment to non-hospitalized patients who tested positive for COVID-19 and were at high risk of progression to severe disease. At the time of auditing (November 2022), NHS England had commissioned four different treatment options. Even though each of them was not directly compared in clinical trials at the time, NHSE clearly specified the order in which treatment option should be considered. Nirmatrelvir boosted with ritonavir or sotrovimab was first line. Then, remdesivir and finally molnupiravir are the last options.^1,2,3^ Even though molnupiravir has less drug–drug interactions and can be used with patients in all stages of renal impairment, NHSE recommended it last because at the time, it appeared to be the least effective of all four therapies.^3^

**Objectives:**

To review current prescribing practice of COVID-19 medicines in the virtual hospital and to improve stewardship within the Acute Medical Unit virtual hospital.

**Standards:**

(i) 100% of patients prescribed molnupiravir had a confirmed SARS-CoV-2 infection, (ii) 100% of patients prescribed molnupiravir were ineligible for treatment with nirmatrelvir/ritonavir due to severe liver impairment or kidney disease, (iii) 100% of patients prescribed molnupiravir were ineligible for treatment with nirmatrelvir/ritonavir due to significant drug–drug interactions and (iv) 100% of patients prescribed molnupiravir should have a documented reason in EPR as to why they were ineligible for treatment with sotrovimab. The criteria and standards above were set using the Interim Clinical Commissioning Policy: Antivirals or neutralizing monoclonal antibodies for non-hospitalized patients with COVID-19.

**Methods:**

All patients admitted to the virtual hospital via the CMDU pathway during the 9-month period from 1 January to 30 September 2022 were included in audit. A total of 602 patients were prescribed molnupiravir during this period, and 34 patients were selected randomly. The data collection involved a retrospective case note review of 34 electronic patient records (EPR) over one working week between 24 October and 28 October 2022. Data from 3 patients were excluded from the audit as liver and/or function tests were unavailable in EPR; thus, *N* = 31. The data collection was recorded on a Microsoft Excel data toolkit, which included the confirmation of SARS-CoV-2 infection, date of treatment commenced, nirmatrelvir/ritonavir drug interactions^1^, liver and renal function tests^2–3^ (ALT, ALP, bilirubin, eGFR/CrCl and CKD) and consideration of sotrovimab as alternative first-line treatment with documented reason. The data was then analysed and graphs were generated to draw results. All standards were set at 100%. This study did not require ethical approval.

**Results:**

The results after data collation revealed that 100% patients prescribed molnupiravir had a confirmed SARs-CoV-2 infection; 29% patients were ineligible for treatment with nirmatrelvir/ritonavir due to stage 3–5 CKD; 29% patients were ineligible for treatment with nirmatrelvir/ ritonavir due to significant drug–drug interactions; 22.6% patients had a documented reason in their electronic record as to why they were ineligible for treatment with sotrovimab.

**Discussion and action plan:**

Majority (71%) of patients prescribed molnupiravir did not have severe CKD or severe liver impairment. They were eligible for treatment with nirmatrelvir/ritonavir, provided that these patients did not have significant drug interactions with concomitant medications. Although most cases had documented the interactions with concomitant medications, 64.5% patients had potential interactions that could have been worked around. Documentation was poor as to why sotrovimab was not considered as first-line alternative treatment and this was fed back to the virtual hospital team to document rationale for treatment going forward. Nirmatrelvir/ritonavir could have been co-administered with concomitant medications in most cases. The team now have access to a dedicated antimicrobial pharmacist when they need advice.

**Limitations:**

Both liver function tests and renal function tests were unavailable at the time when molnupiravir treatment was commenced. Hence, lab results within 1 year were used as part of this audit. Due to time constraints, only a small part of patients prescribed molnupiravir was studied.

**Conclusions:**

Majority of patients were inappropriately prescribed molnupiravir, as their renal function/liver function was not poor enough to exclude them from treatment with nirmatrelvir/ritonavir. The poor documentation means we do not know why the other first-line and second-line options were not considered. Whilst a lot of patients were on medicines that interacted with nirmatrelvir/ritonavir, the majority were not significant and could have been worked around.
